# Data quality of the routine health management information system at the primary healthcare facility and district levels in Tanzania

**DOI:** 10.1186/s12911-020-01366-w

**Published:** 2020-12-17

**Authors:** Susan F. Rumisha, Emanuel P. Lyimo, Irene R. Mremi, Patrick K. Tungu, Victor S. Mwingira, Doris Mbata, Sia E. Malekia, Catherine Joachim, Leonard E. G. Mboera

**Affiliations:** 1 National Institute for Medical Research, Headquarters, Dar es Salaam, Tanzania; 2 SACIDS Foundation for One Health, Sokoine University of Agriculture, Morogoro, Tanzania; 3 National Institute for Medical Research, Amani Research Centre, Muheza, Tanzania; 4 Ministry of Health, Community Development, Gender, Elderly and Children, Dodoma, Tanzania

**Keywords:** Health information, Data, Quality, Indicators, Completeness, Accuracy, Facility, District, Tanzania

## Abstract

**Background:**

Effective planning for disease prevention and control requires accurate, adequately-analysed, interpreted and communicated data. In recent years, efforts have been put in strengthening health management information systems (HMIS) in Sub-Saharan Africa to improve data accessibility to decision-makers. This study assessed the quality of routine HMIS data at primary healthcare facility (HF) and district levels in Tanzania.

**Methods:**

This cross-sectional study involved reviews of documents, information systems and databases, and collection of primary data from facility-level registers, tally sheets and monthly summary reports. Thirty-four indicators from Outpatient, Inpatient, Antenatal care, Family Planning, Post-natal care, Labour and Delivery, and Provider-Initiated Testing and Counselling service areas were assessed. Indicator records were tracked and compared across the process of data collection, compilation and submission to the district office. Copies of monthly report forms submitted by facilities to the district were also reviewed. The availability and utilization of HMIS tools were assessed, while completeness and data accuracy levels were quantified for each phase of the reporting system.

**Results:**

A total of 115 HFs (including hospitals, health centres, dispensaries) in 11 districts were involved. Registers (availability rate = 91.1%; interquartile range (IQR) 66.7–100%) and report forms (86.9%; IQR 62.2–100%) were the most utilized tools. There was a limited use of tally-sheets (77.8%; IQR 35.6–100%). Tools availability at the dispensary was 91.1%, health centre 82.2% and hospital 77.8%, and was low in urban districts. The availability rate at the district level was 65% (IQR 48–75%). Wrongly filled or empty cells in registers and poor adherence to the coding procedures were observed. Reports were highly over-represented in comparison to registers’ records, with large differences observed at the HF phase of the reporting system. The OPD and IPD areas indicated the highest levels of mismatch between data source and district office. Indicators with large number of clients, multiple variables, disease categorization, or those linked with dispensing medicine performed poorly.

**Conclusion:**

There are high variations in the tool utilisation and data accuracy at facility and district levels. The routine HMIS is weak and data at district level inaccurately reflects what is available at the source. These results highlight the need to design tailored and inter-service strategies for improving data quality.

## Background

Disease prevention and control requires prompt and adequate actions towards reduction or elimination of existing conditions, and preventing new occurrences. Efficient decisions to such actions should be based on correctly collected, analysed, interpreted and timely data. In low- and middle-income countries, data for decision-making are generated by the health information systems, mostly through the routine Health Management Information System (HMIS) [1]. HMIS integrates data collection, processing, reporting and facilitates use at all levels to improve health service effectiveness and efficiency in response [1, 2]. HMIS collects data at health facilities (HFs), which contains statistics on health services, disease epidemiology, and administration [3]. Quality information is essential to monitor, evaluate, prioritize, and improve the delivery of health care services [1, 2, 4].

Despite the fact that the HMIS is the backbone for strong health systems, studies in Sub-Saharan Africa (SSA) have reported challenges with data quality, including completeness and timeliness, accuracy, consistence and poor utilization of HMIS tools [1, 5–13]. The concerns about the quality of routine information have undermined data utilization for decision-making in the health sector [9, 10, 14–20]. Completeness and timeliness entails completeness of reports, completeness of data and timeliness of reports; while consistency refers to accuracy, outliers, trends and consistency between indicators. A recent study in Ethiopia, found that completeness and timeliness ranged from as low as 32% to as high as 75% of the facility reporting [20]. Another study in Nigeria, reported that facility-reported data were incomplete by 40% of the time [18]. On the other hand, internal data inconsistency is quite common in a number of countries in Sub-Saharan Africa [18, 19, 21]. Both under- and over-reporting have been frequently observed, and it varied across indicators, facilities and districts [18, 22]. For instance, in a study in Rwanda, over-reporting was observed for ante-natal care-related data than for other indicators [19]. In some cases, missing values, measurement error, inaccuracy and false reports from unidentified sources have been observed [20]. Under-reporting of the levels of 10–60% at facility level have been reported in Nigeria [18].

Challenges in data quality in SSA are compounded by human, health system and infrastructure factors [1, 2, 16]. Healthcare workers face a poor understanding of HMIS tools and the variables/indicators, inadequate skills, workload, and lack of incentives [9, 11, 13]. Excessive data demand, large number of reports, frequent changes in HMIS tools, changes in organisation structures or of human resources, lack of effective systems to monitor quality and absence of standards guidance to measure data quality contribute to poor quality [16, 17]. Limited infrastructure and means to transmit reports from one level to another add more complexity [5, 14, 17–19]. Data quality assessments need to address these attributes and processes to establish valid conclusions that foster solution-focused thinking [15, 16].

Weak health information systems in SSA are critical challenges to reaching the global and national health goals because the health system performance cannot be adequately monitored where data are of poor quality [2, 10]. It is evident that increased investment in health is dependent on an efficient and reliable HMIS. With the current increased investment in disease control, availability of quality information is critical. Significant efforts have been made to strengthen health information systems and improve the quality of data used for decision-making at different levels of the health system. Following introduction of electronic health information systems quality indicators such as reporting completeness and timeliness have significantly improved in many countries [16, 20, 23]. We hypothesize that, despite improvement on these attributes, the accuracy level of the data reported is not adequate. In Tanzania, available evidences suggest lack of HMIS data quality [9, 24–26]; and that of recently, no robust assessment and analysis at primary health care and district levels has been done. This study, therefore, assessed the quality of the routine HMIS data at primary health care facility and district levels in Tanzania focusing at the utilization of the tools used for capturing data and consistency of records from the original source (health facility register) to the final point (national level) to determine attribute-based differentiation of quality levels and propose strategies for improvement.

## Methods

### Study design

**Fig. 1:**
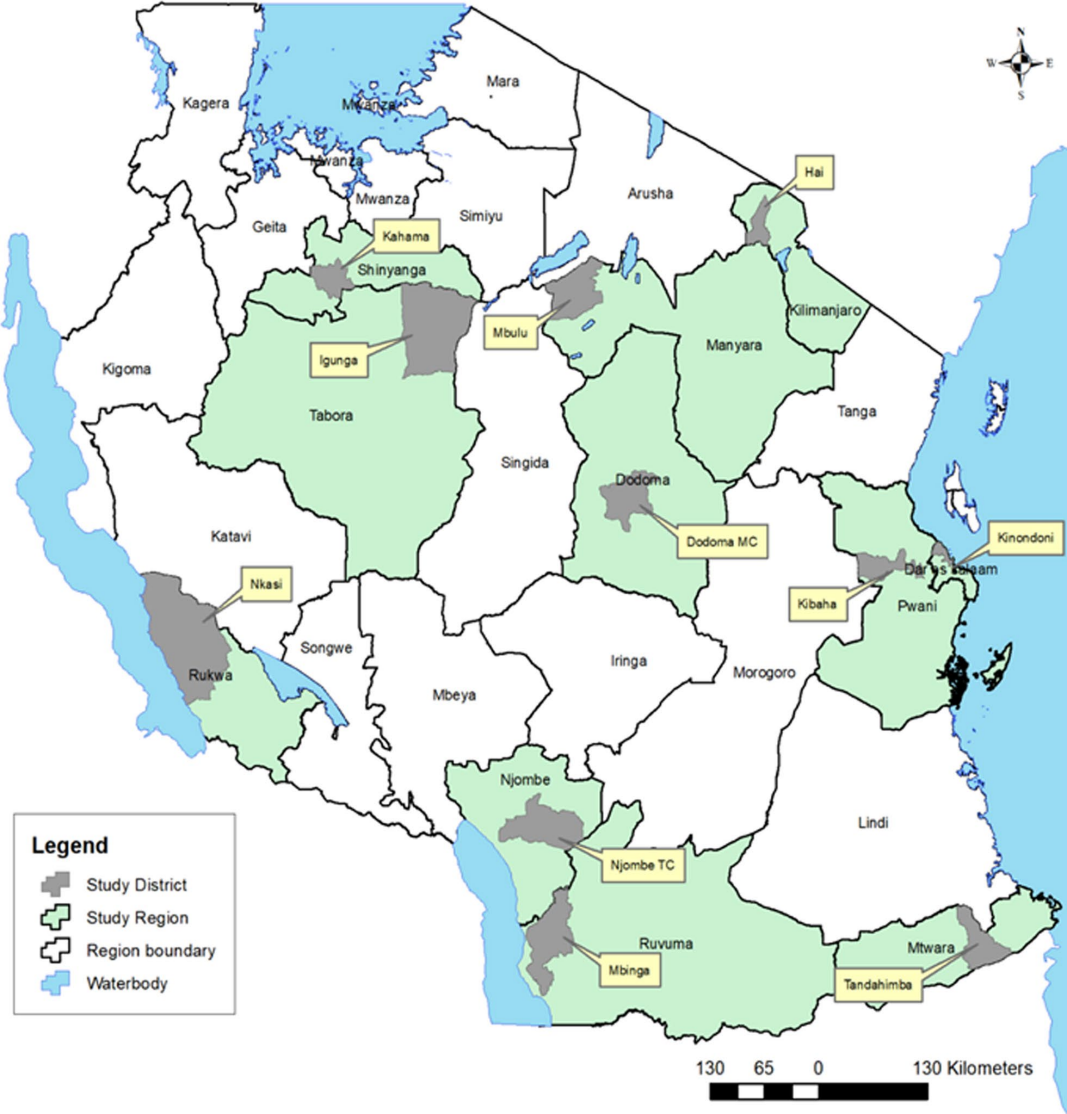
Map of Tanzania showing the study regions and districts (This is an original map generated by the authors)

This cross-sectional study was carried out from October–November 2017 and involved 11 districts and all levels of primary health care facilities (i.e. dispensary, health centre, and hospital) in Tanzania (Fig. 1). A multistage sampling technique was used to randomly select 1–2 regions from each geographical zone [27] and one district from each region. List of facilities per district (registered and functional) were obtained from the Health Facility Registry (http://moh.go.tz/hfrportal). District hospitals were conveniently included. However, where there was no district hospital, a regional hospital was included. At least 50% of the health centres (HC) with a minimum–maximum criterion of 2–4, and 20% of dispensaries (min–max of 5–8) were randomly selected.

### Data sources

The study involved reviews of documents, information systems and databases and collection of primary data at facility and district levels. The source of data included facility-level registers, tally sheets and monthly summary reports (paper and electronic). Seven service areas namely Outpatient department (OPD), Inpatient department (IPD), Antenatal care (ANC), Family Planning (FP), Post-natal care (PNC), Labour and Delivery (LnD) and Provider-Initiated Testing and Counselling (PITC) were included in the assessment.

A total of 34 indicators selected from each of the service area (OPD = 5; IPD = 4; ANC = 8; PNC = 6; LnD = 2; FP = 6; PITC = 3) were assessed. The age category for the indicators followed the grouping used for reporting in HMIS tools. The selected indicators for OPD and IPD (both with age categorised as < 5 and ≥ 5 years) were anaemia (mild, moderate, severe) and malaria (confirmed malaria blood slide positive, confirmed malaria blood slide, clinical malaria). For the ANC the indicators were pregnancy (< 12 and ≥ 12 weeks of pregnancy, pregnant mothers who received tetanus toxoid, pregnant women who received intermittent preventive treatment for malaria, pregnant women who received first test of HIV, and mothers who tested positive for HIV during first test. The indicators for PNC were the delivered mothers who attended clinic within 48 h; who completed all the visits; who were diagnosed with severe anaemia; who acquired mental illness; and those who chose exclusive breast feeding. Indicators for LnD were women who delivered at health facility and those who delivered with the assistance of skilled personnel. There were 6 indicators for FP, namely number of clients for injection methods, number of clients receiving pills at health facility, inserting inter-uterine device, those screened for breast cancer, and those screened for cervical cancer. Indicators for PITC were the number of new clients, number of new clients tested positive for HIV and number of clients returned for counselling after testing. The selected indicators included those that were easy to collect, difficult to understand, difficult to compile, and takes time to compile.

The research team for each district comprised of four trained research assistants under the supervision of two senior researchers. Training was done before actual data collection and involved pilot exercise to ensure clarity on the HMIS tools (registers, tally sheets and reporting forms) and how they are supposed to be used, the type of data to be collected, as well as ethical issues when dealing with patient information.

### Data collection procedures

**Fig. 2:**
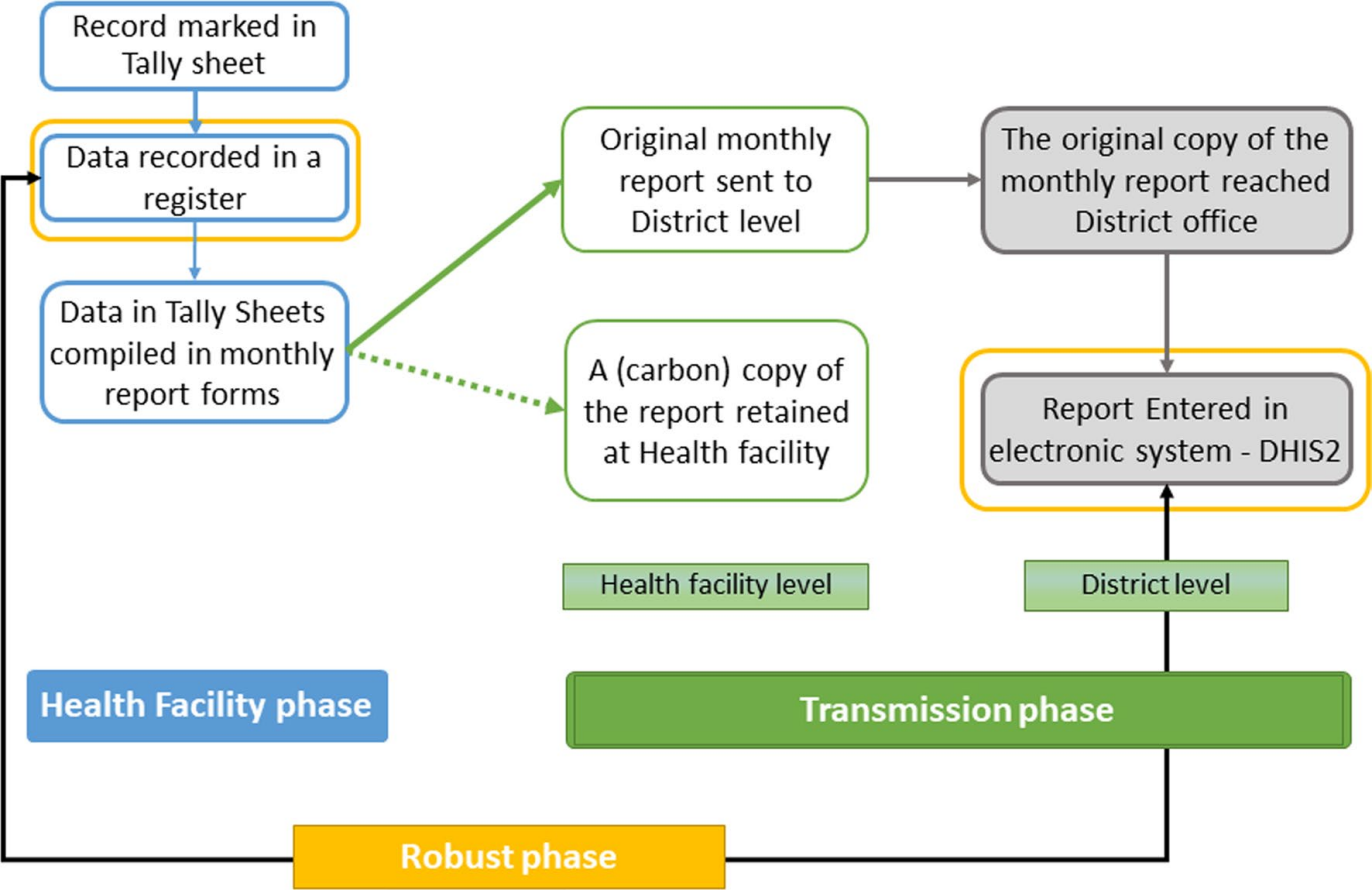
The routine HMIS data journey

The assessment considered and tracked data based on the order of events in the existing HMIS (referred to in this context as “data journey”). Primarily, patient data are recorded in registers at the time a client is been attended. The records are compiled at the end of each month to make a report, done in duplicate and separately for each service area. Tally sheets, designed for each service area with the same structure as the reported indicators, are used daily to track each record. The original monthly report is submitted to the district office. Used registers, tally sheets and carbon copies of all reports are kept at the facility for their use and future reference. The reports submitted to the district office are expected to be filed and organized and the data is later entered in an electronic system known as District Health Information Systems (DHIS2) for further analysis and use (Fig. 2).

At the facility, records of each indicator were tracked across all sources, i.e. physical counts from registers, records marked in tally-sheets and compiled totals in report forms. At the district office, original copies of monthly report forms submitted by facilities were reviewed. The filled records were compared to those observed in the carbon copies of reports (found at the facility), and to what has been entered in the DHIS2. The period of data assessment was January 2014 to September 2017 (45 months) with a detailed review covering 12 months. To select months for the detailed review, we fixed the last quarter of 2014 (October–December) and the first quarter of 2017 (January–March) (providing maximum time to resolve existing data issues). Then we conveniently assigned April–June 2016 and July–September 2015 to get an equal time interval (6 months) between the quarters.

### Definition of indicators

At the facility level, tool availability was defined as the presence of filled/used HMIS tools (registers/tally-sheets/report forms by service area). At the district office, we tracked report forms from the health facilities for the selected 12 months. This tracking aimed to establish the fact that patient records were filled, tally-sheets were utilized, reports were compiled and submitted to the district office each month. The availability rate at the facility level was calculated as the percentage of observed registers/tally-sheets/reports out of those expected in a specified period. The availability rate at the district level was used to measure reporting completeness (percentage of reports received from HFs in a specified period). Completeness focused on recording, by reviewing the filling practices of registers compared to the provided instructions. Accuracy measured the numerical correspondence between data reported in one tool (subsequent) and that appearing in the source.

### Data management and analysis

Data entry was done in EpiData 3.1 (EpiData Association, Odense M, Denmark), then migrated to STATA 13 (STATA, College Station, TX) for analysis. Quality check was done by comparing a random pick of 10% of entered data with the one in the original forms. We assessed data quality by year, service area, indicators, tool, facility level, ownership and district. Based on the total expected register-months, tally sheet-months and monthly reports, we calculated the median availability rates and the interquartile range (IQR). For the IQR we presented the 25th and 75th percentiles, and range (p75–p25) to assess variability in performance.

For accuracy, we calculated a difference ratio (an index measure that quantifies the difference between data sources) [8, 15, 18]. Six difference ratio (DR) indices, *Diff1–6*, were calculated, assessed, and grouped considering the three phases of the *data journey*, i.e. from the point a record is entered in a register at the facility to the point it reaches the DHIS2. The “health facility phase” focused on data activities occurring at the healthcare facility (recording to report compilation). The “transmission phase”, focused on report processing and communication (facility-district, district files-DHIS2) accounting for the revisions/corrections happening during this process. The “robust phase” compared data recounted from registers with totals entered in the DHIS2. With this phase, we present an *ideal situation* where all quality issues at the facility or during transmission are masked and data of the two extreme ends of the *data journey* is compared. This categorization facilitates systematic understanding, tagging quality issues and explore mechanisms to design practical-oriented data quality interventions stage wise.

DR < 1 indicates fewer data in the subsequent source, implying under-representation. DR > 1 means more data in the subsequent source, implying over-representation, while DR ≈ 1 implying consistency between sources. Matching levels were categorized into 5 groups (depending on the increase/decrease from DR = 1) and presented using colour-coded tables by different attributes. These were: (1) matched: 0.95 ≤ DR ≤ 1.05, i.e. acceptable difference  ± 5%; (2) moderately matched: 0.75 ≤ DR < 0.95 or 1.05 < DR ≤ 1.25; (3) moderately under-represented: 0.5 < DR ≤ 0.75 or moderately over-represented: 1.25 < DR ≤ 1.5; (4) highly under-represented: DR < 0.5 or highly over-represented: 1.5 < DR ≤ 2; and, (5) extremely over-represented: DR > 2. Statistical significance was tested using *t* tests and proportional tests, significance considered at *p* value < 0.05.

## Results

### Tools availability

A total of 115 healthcare facilities in 11 districts were assessed. Both urban and rural districts were included in the study. The urban districts (and the number of facilities) were Dodoma (11), Igunga (10), Kahama (10), Kinondoni (18) and Njombe (10). The rural districts were Hai (10), Kibaha (8), Mbinga (10), Mbulu (8), Nkasi (10) and Tandahimba (10). Dodoma had no district hospital, therefore, the respective regional hospital was included. Due to a large number of private facilities in Kinondoni district, additional private hospital and two private health centres were included. Of the 115 HFs, 58.3% (n = 67) were dispensaries, 31.3% (n = 36) health centres and 10.4% (n = 12) hospitals. Of all the HFs, 114 had OPD, IPD (43), ANC (108), PNC (105), PITC (94), LnD (93) and FP (88) service-areas.

**Fig. 3:**
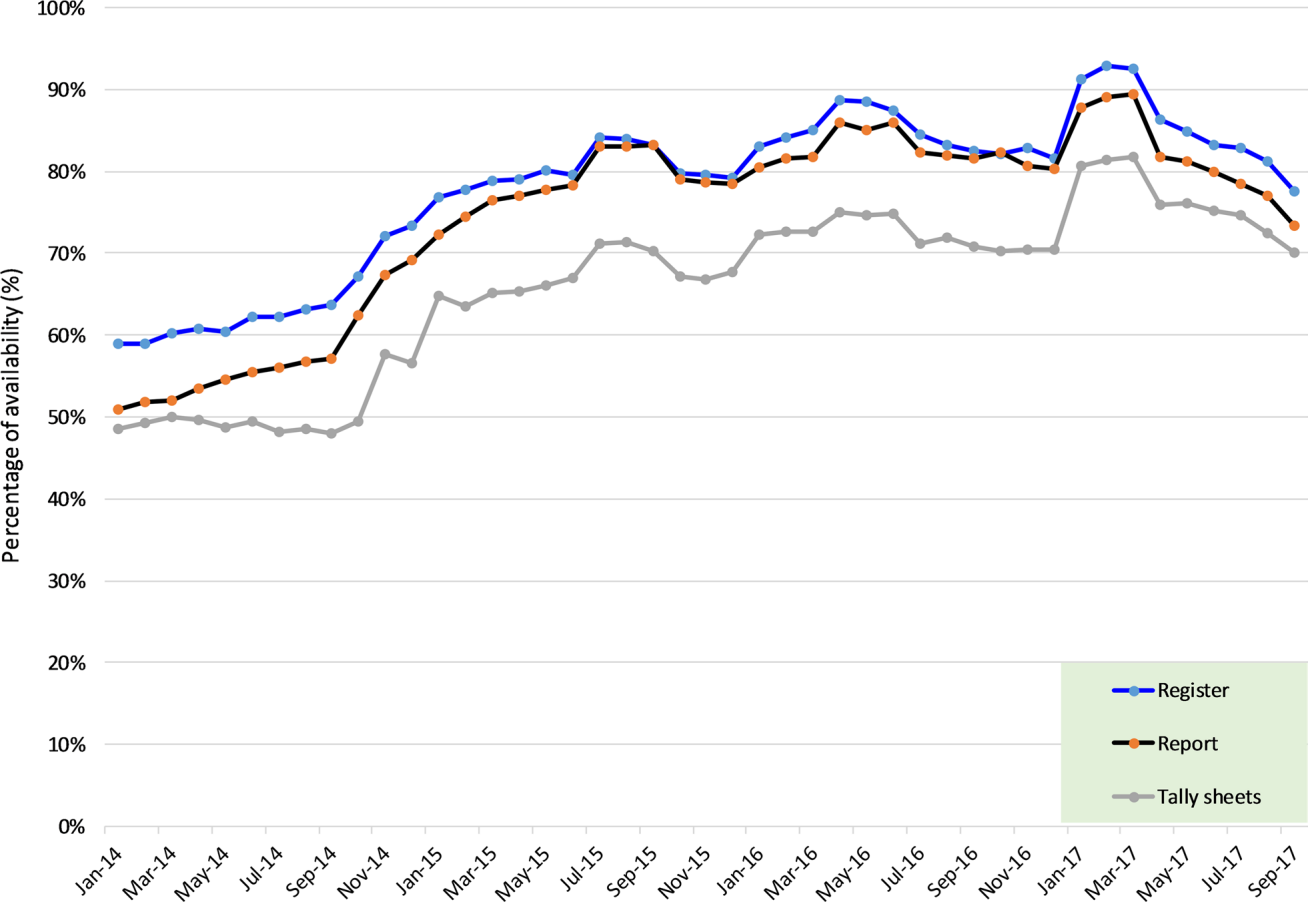
Overall trends in the availability of HMIS tools by type

The overall median availability rate for registers was 91.1% (IQR 66.7%, 100%) compared to 77.8% (IQR 35.6%, 100%) and 86.9% (IQR 62.2%, 100%) for the tally-sheets and report forms, respectively (Table 1). HMIS tools were mostly available at the dispensaries 91.1% (IQR 60%, 100%) than health centres 82.2% (IQR 55.6%, 100%) and hospitals 77.8% (IQR 30%, 97.8%) (*p* value < 0.0001). Faith-based owned facilities had a significantly higher amount of tools available than the government and private-owned facilities (*p* value < 0.0001). The service-areas with high tool availability rates were ANC 95.6% (IQR 73.3%, 100%), FP 93.3% (IQR 66.7%, 100%) and LnD 91.1% (IQR 73.3%, 100%). PITC had the lowest rate 53.3% (IQR 20%, 88.9%). Hai, Kibaha, Mbinga, Mbulu (rural districts) had the highest availability rates while Kinondoni and Dodoma (urban districts) had the lowest (Table 1). A high variation in the range value was observed in the tally-sheet, indicating a significant difference in its utilisation between facilities. A remarkable increasing trend in the availability of tools with lesser variation between HFs was observed from 2014 (median = 83.3%; range = 100%) to 2017 (median = 100%; range = 22%) (Table 1; Fig. 3).

**Table 1:** Status of overall HMIS tool availability rates at HF by different attributes

Variable	Categories	Median (%)	IQR (p25,p75)	Range (%)	*p* value
All years	Registers	91.1	(66.7,100)	33	< 0.001
	Report forms	86.7	(62.2,100)	38	
	Tally-sheets	77.8	(35.6,100)	64	
2014	Overall	83.3	(0,100)	100	
	Registers	91.7	(16.7,100)	83	
	Report forms	75.0	(0,100)	100	
	Tally-sheets	41.7	(0,100)	100	
2015	Overall	100.0	(50,100)	50	
	Registers	100.0	(75,100)	25	< 0.001
	Report forms	100.0	(75,100)	25	
	Tally-sheets	100.0	(16.7,100)	83	
2016	Overall	100.0	(83.3,100)	17	
	Registers	100.0	(91.7,100)	8	
	Report forms	100.0	(91.7,100)	8	
	Tally-sheets	100.0	(33.3,100)	67	
2017	Overall	100.0	(77.8,100)	22	
	Registers	100.0	(88.9,100)	11	
	Report forms	100.0	(77.8,100)	22	
	Tally-sheets	100.0	(55.6,100)	44	
Facility level	Dispensary	91.1	(60,100)	40	< 0.001
	Health centre	82.2	(55.6,100)	44	
	Hospital	77.8	(30,97.8)	68	
Facility Ownership	Faith-based organization	91.1	(68.9,100)	31	< 0.001
	Government	86.7	(60,100)	40	
	Private	68.9	(20,95.6)	76	
Service area	Antenatal care	95.6	(73.3,100)	27	< 0.001
	Family planning	93.3	(66.7,100)	33	
	Inpatient	77.8	(44.4,100)	56	
	Labour and delivery	91.1	(73.3,100)	27	
	Outpatient	83.3	(57.8,100)	42	
	PITC	53.3	(20,88.9)	69	
	Post-natal care	77.8	(46.7,97.8)	51	
District	Dodoma	60.0	(24.4,88.9)	64	< 0.001
	Hai	97.8	(71.1,100)	29	
	Igunga	87.8	(66.7,100)	33	
	Kahama	80.0	(55.6,100)	44	
	Kibaha	93.3	(77.8,91.1)	13	
	Kinondoni	46.7	(22.2,80)	58	
	Mbinga	95.6	(75.6,100)	24	
	Mbulu	100.0	(91.1,100)	9	
	Njombe	88.9	(71.1,100)	29	
	Nkasi	78.9	(66.7,97.8)	31	
	Tandahimba	88.9	(60,100)	40	

*PITC* provider-initiated testing and counselling

**Fig. 4:**
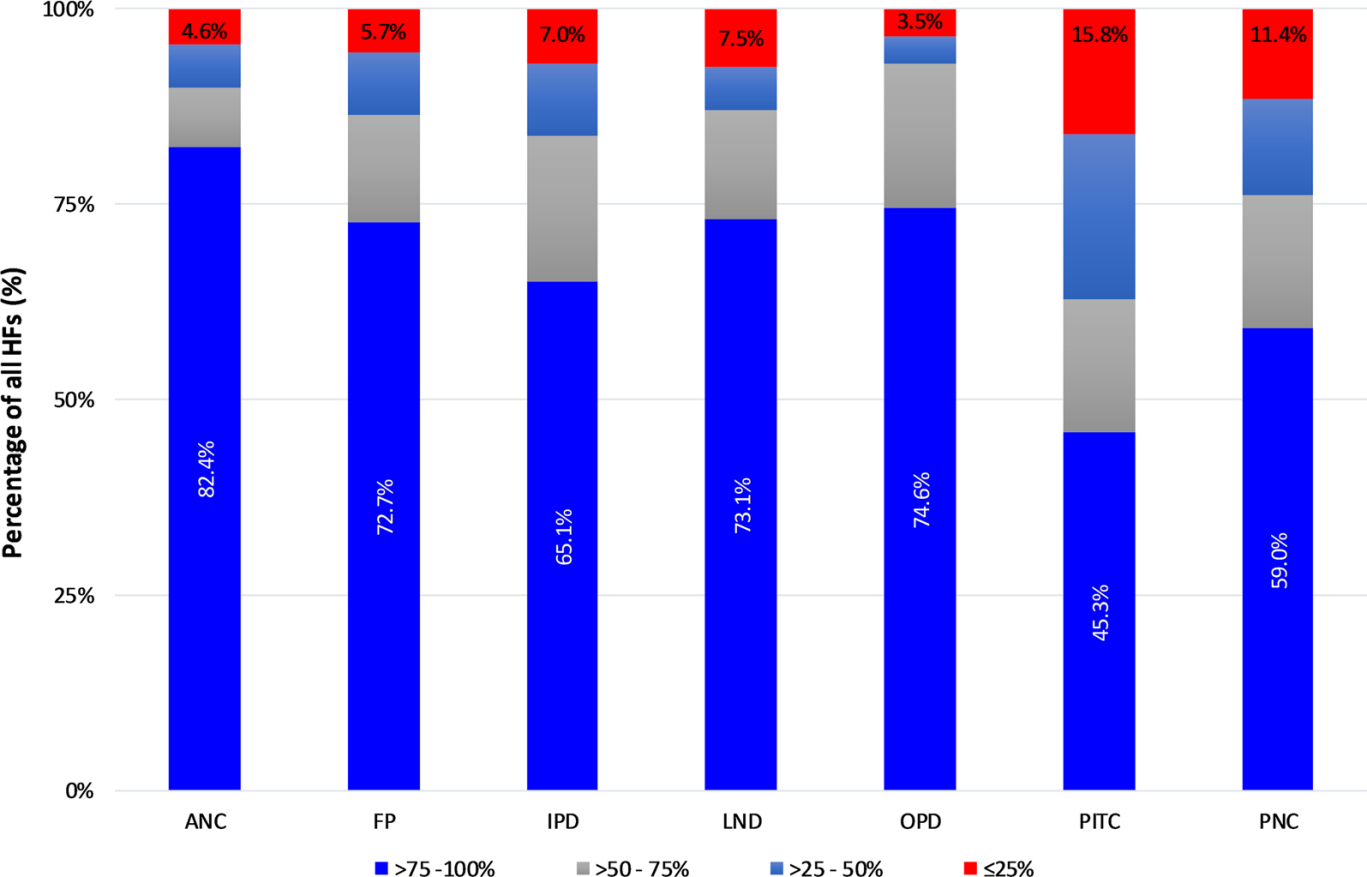
Register availability rates by service area

**Fig. 5:**
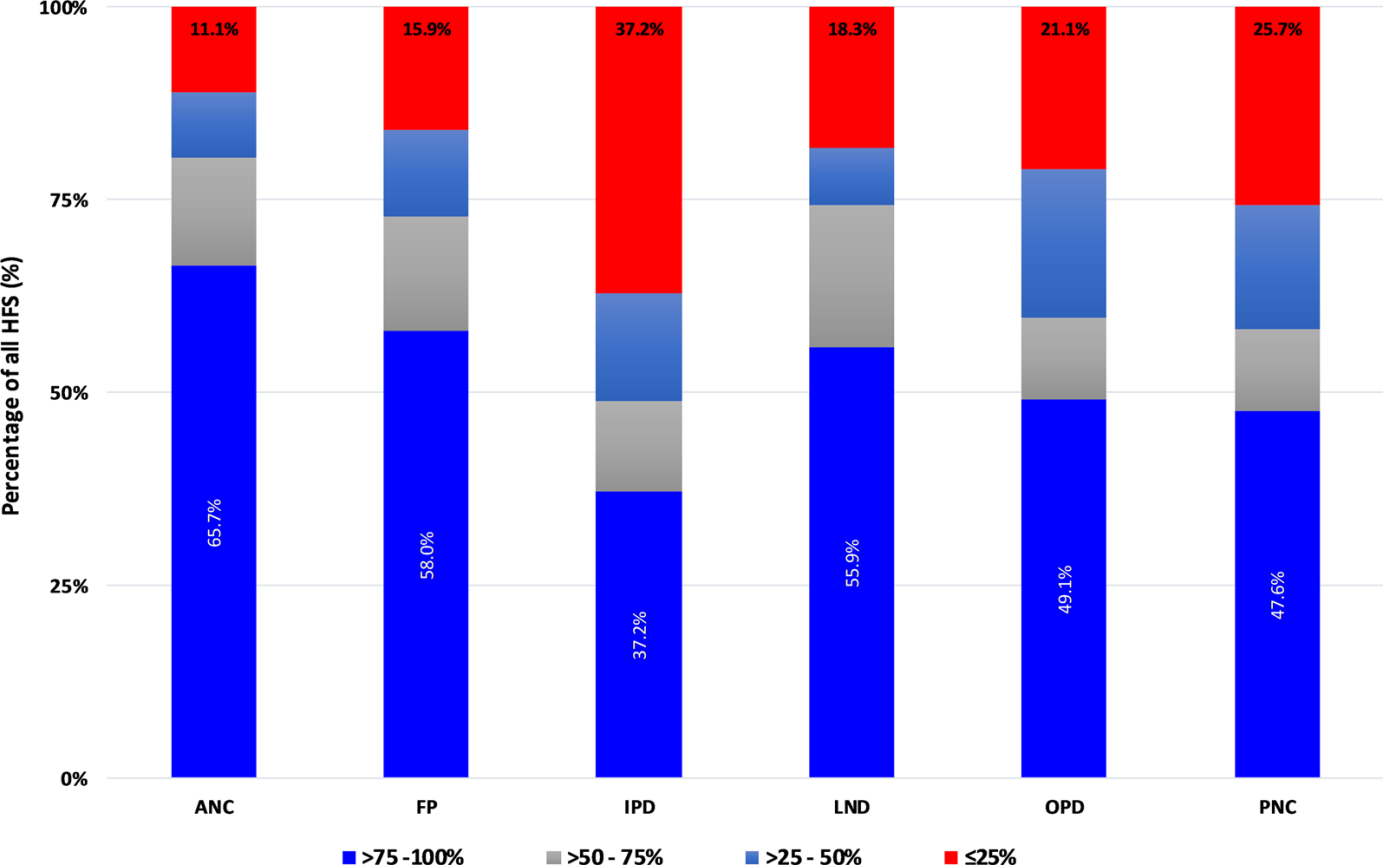
Tally-sheet availability rate by service area

**Fig. 6:**
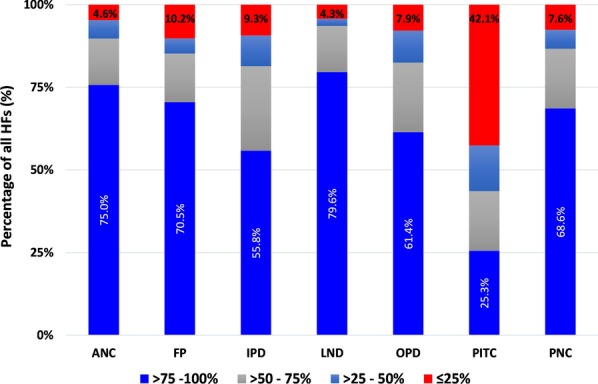
Report availability rate by service area

In terms of availability of tools, we categorised HFs into 4 groups: (1) > 75–100% (very high); (2) 50–75% (high); (3) 25–50% (average); and (4) < 25% (low). In all service areas, with exception of PITC, over 50% of the facilities were able to locate up to > 75% of the required registers (Fig. 4), with high percentages observed in the ANC (82.4%), OPD (74.6%), LnD (73.1%) and FP (72.7%) service areas. PITC registers were rarely available with only 45% of the facilities been able to locate > 75%. Over 15% of the facilities presented less than 25% of the expected PITC registers followed by PNC service-area (11.4%). Tally-sheets for ANC, LnD, and FP were available in larger proportions than those for IPD, OPD, and PNC (Fig. 5). Report forms were highly available in all service-areas except for PITC which had 42.1% of facilities providing ≤ 25% of expected report forms (Fig. 6). Urban districts of Igunga, Kibaha, Njombe, Kinondoni, and Tandahimba fell into average or low categories of tool availability particularly on tally-sheets.

**Fig. 7:**
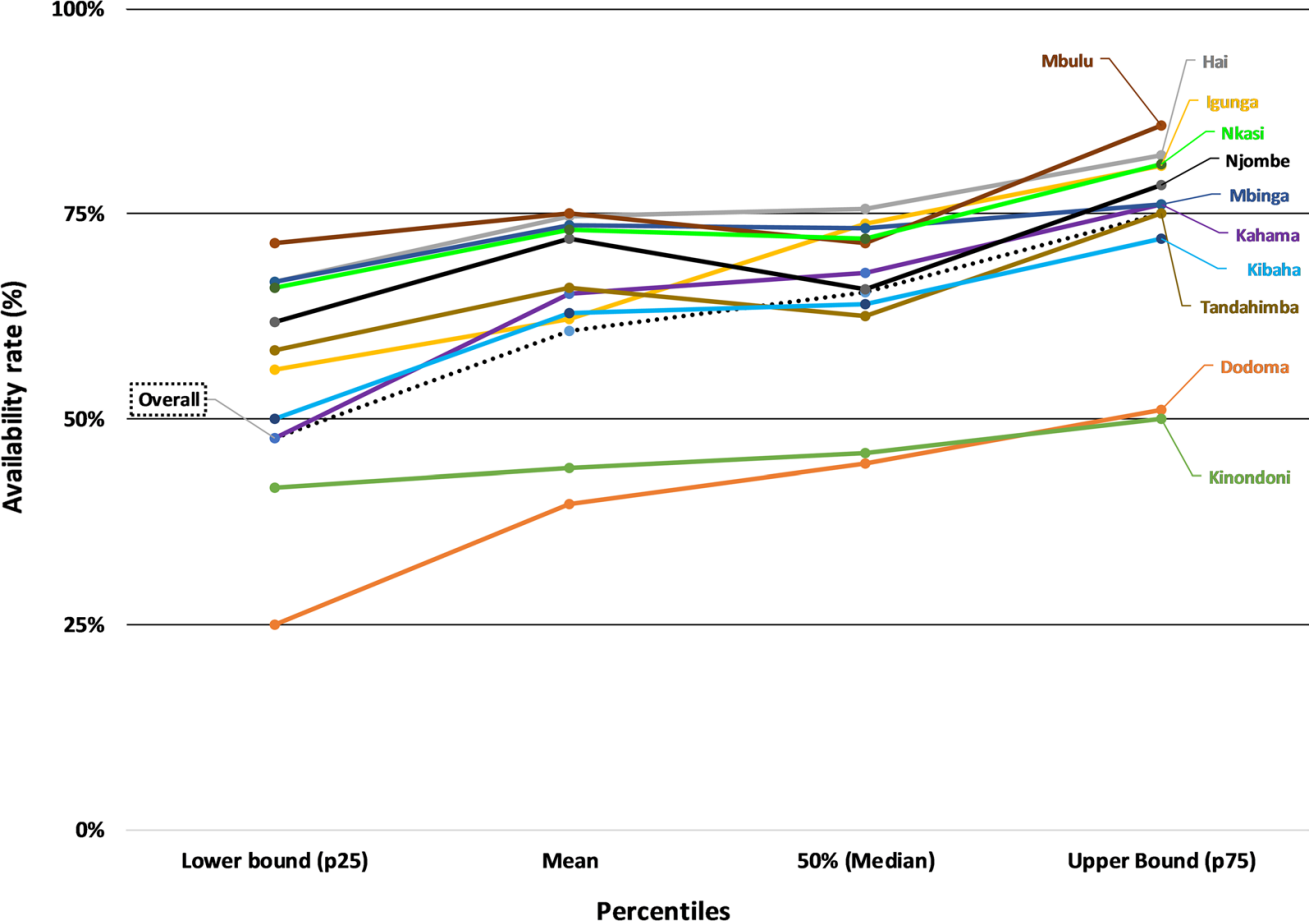
Median, mean and IQR of report availability at the district office

**Fig. 8:**
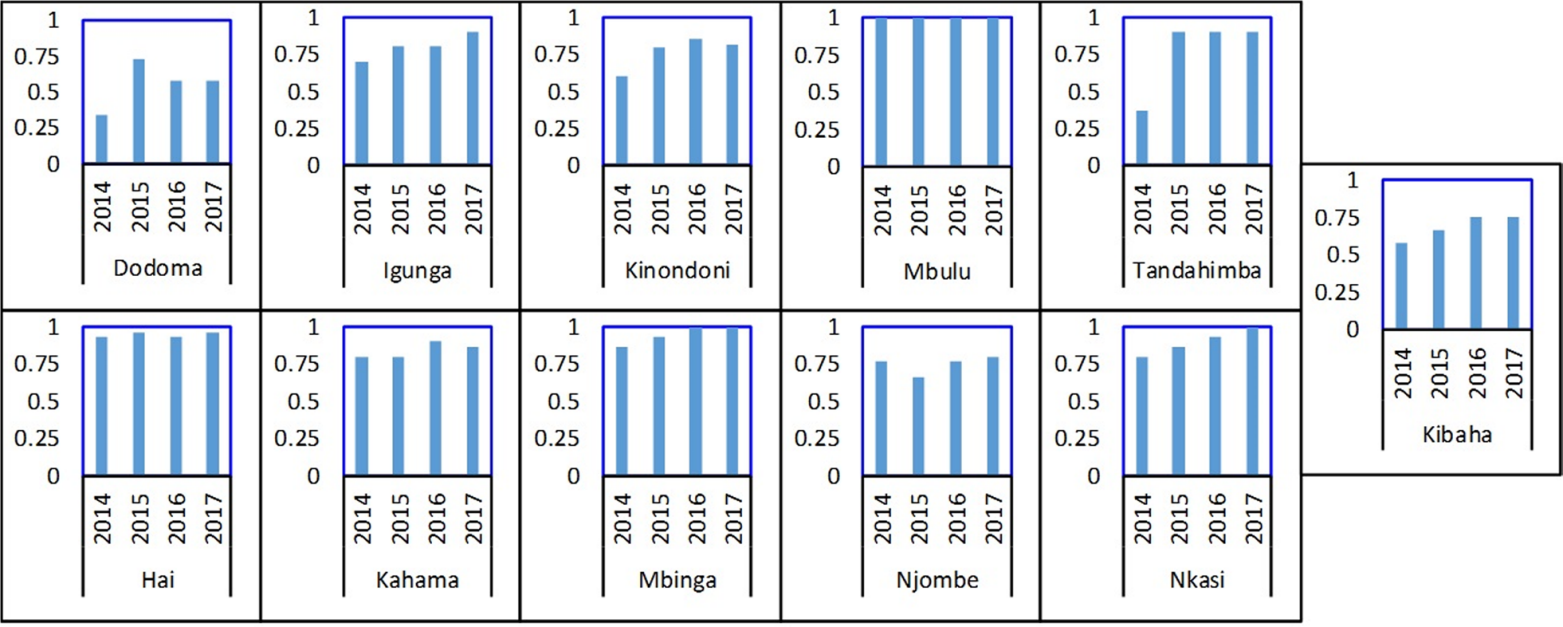
Report form availability rate at the district office

At the district office, the overall median availability of submitted HF reports was 65% (IQR 48%, 75%) indicating that a third of expected report forms were not found. The district-specific performance indicated that less than half of the expected reports were found in the urban districts of Dodoma (median = 45%, IQR 25%, 51%) and Kinondoni (median = 46%, IQR 41–50%) (Fig. 7). Rural districts had higher rates: Hai (75%, IQR 67%, 82%), Igunga (74%, IQR 56%, 81%), Mbinga (73%, IQR 67%, 76%), Nkasi (72%, IQR 66%, 81%), and Mbulu (71%, IQR 71%, 86%). We matched availability of reports at district level and HF by service area for each of the four quarters included in the 12-month detailed review period. The findings indicate higher availability of reports at the HF level than at the district level with variations between service areas and HFs. Reproductive health service areas (PNC, LnD, ANC) performed better than other service areas. The PITC reports were difficult to find at both levels, especially at district office while the IPD reports were often missing. Challenges in transmission of reports, differences in programme reporting practices and weakness in the filing system at the district office were reported. There was no standard filing system of the received HF reports which hindered assessing if the report was received or not. In most districts, there was an increase in the availability of report forms over the years. However, the availability rate in the urban districts of Dodoma, Kibaha, Kinondoni and Njombe remained low during the period under review (Fig. 8).

### Completeness

Wrongly filled or empty cells in HF registers were common. Diagnoses were either not recorded or recorded without indicating disease severity (as instructed) or without laboratory results when available. This was common for malaria and anaemia. In the OPD registers, it was a common practice for patient’ height and weight variables to be left blank, and occasionally sex and age were not filled.

Poor adherence to the coding procedures was frequent. For instance, instead of using “*N-Ndiyo*” and “*H-Hapana*” (Kiswahili words for “Yes” and “No”, respectively), several records were in the English version of the words “*Y-Yes*” and “*N-No*”. In other situations, instead of using a “tick” mark as instructed when the service was provided, a recorder would use “N” or “X”, or leave the entries blank or use a different code that meant a different thing altogether. Consequently, this resulted in changing the meaning of that particular record. In some cases, health workers couldn’t remember the meanings of some of the codes they used. Such practices were reported to complicate compilation of the report, especially if a different person (from the one who did the recording) is compiling the report.

Improper use of carbon papers was observed in HFs, to the extent that it was difficult to identify the value recorded in the report form. The use of worn-out carbon papers was common and resulted into a blank or very faint report copies. Such poor recording practices led to differences between recounted and reported data, hence low accuracy performance.

### Data accuracy

*Antenatal care service (ANC)* At the HF phase, Diff1 indicates over 50% representation of data in tally-sheets while Diff2 shows extreme over-representation (of close to 3-folds) in the reports compared to registers’ records. A similar pattern was observed for Diff6 when registers counts were compared to the DHIS2 records. The transmission phase indicated consistency. The slight difference between Diff2 and Diff6 (with stable Diff3–Diff5) suggests that the reports transmitted to the district were manipulated (corrected/revised) before entered in the DHIS2, yet the changes were not documented. The over-representation levels decreased slightly over the years (Table 2).

**Table 2:**
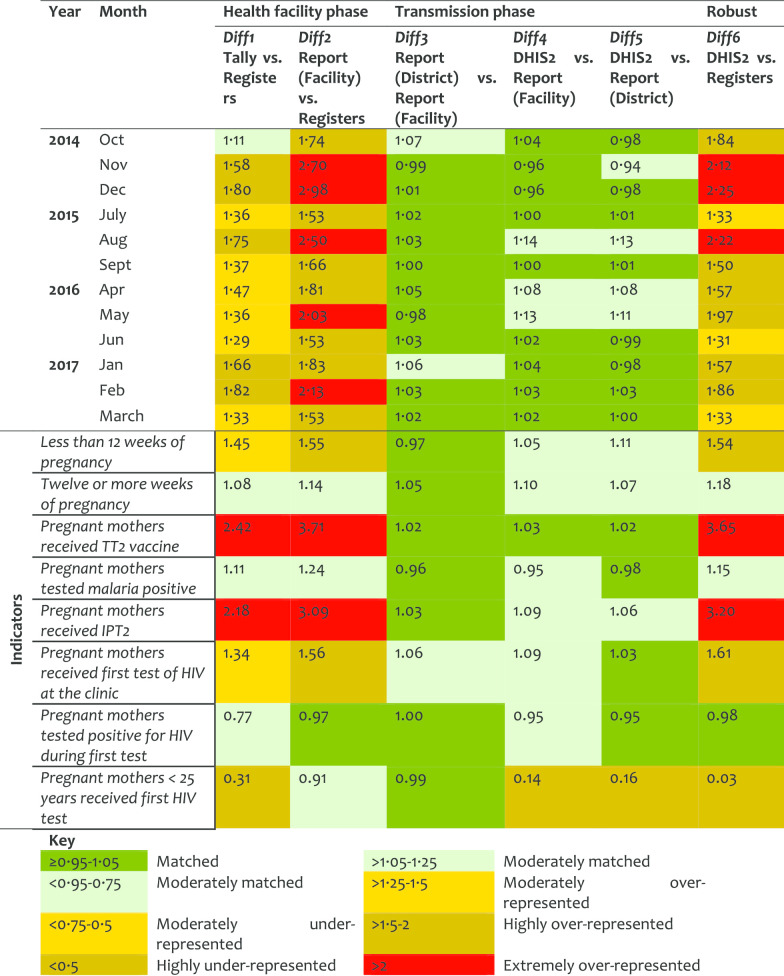
Data accuracy as indicated by difference ratio for the antenatal care service area

The indicators for the provision of tetanus vaccine (TT2) and malaria intermittent preventive treatment (IPT2) performed poorly with lower registration than what had been tallied (> 2-folds), compiled, and reported (> 3-folds). This implies that there was intensive marking of clients in tally-sheets without registration. Indicators on gestation age and HIV testing for pregnant women were moderately matched in the tally-sheets and reports. The indicator for HIV testing among pregnant women < 25 years old was highly under-represented in all phases (Diff1 = 0.31, Diff4 = 0.14; Diff5 = 0.16 and Diff6 = 0.03). Data were found in registers but not reflected in the tally-sheets or report forms or DHIS2. There was variation in the ANC performance by districts in Diff1, Diff2, and Diff6 with much higher over-representation observed in Mbulu, Kinondoni, Kahama, and Nkasi districts.

*Labour and Delivery service (LnD)* At the HF level there was over-representation of data in the tally-sheets and reports as compared to what was recorded in the registers. In 2016, at the transmission phase, there was an over-representation of data in the district report as compared to the copy available at the facilities indicating that the reports were not comparable. There was no significant difference between Diff2 and Diff6. The Diff6 values decreased over time indicating an improvement in the data accuracy (Table 3). The first indicator had good matching levels at the HF phase. However, it was found to be revised at the transmission phase, thus more records were observed in the district copy than in the HF (source) copies, DR = 1.31. For the second LnD indicator, few data were observed in the registers than tally-sheets or reports. Although a large number of clients were indicated to deliver at the HF as marked in the tally sheet, almost none were marked of who assisted in the delivery. The values in a report for this period matched with those of who delivered at the HF. There was a little variation on the data quality performance by district on LnD.

**Table 3:**
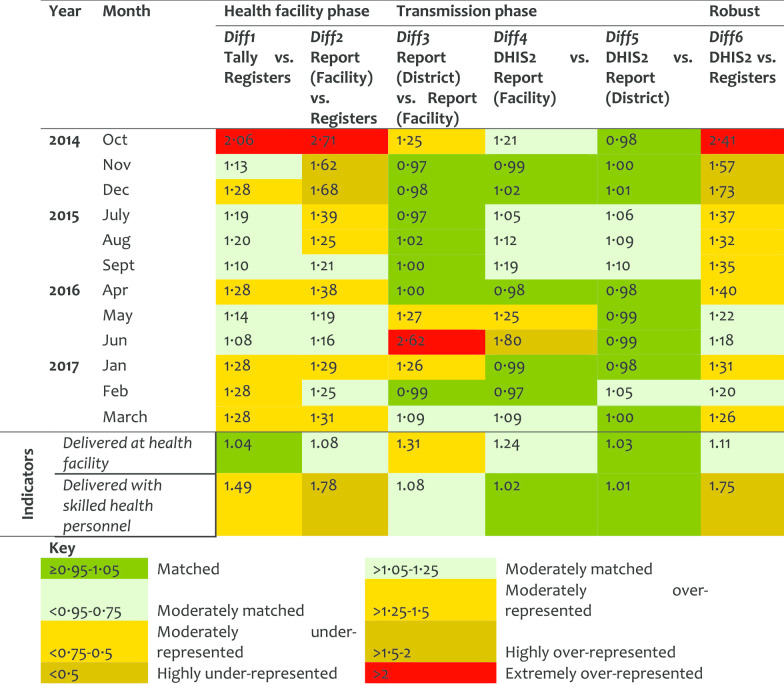
Data accuracy as indicated by difference ratio for the labour and delivery service area

*Post-natal (PNC) service* For the PNC, the quality of data, especially in the filling of tally-sheets and compilation, improved significantly over the years. However, the results indicated that sometimes the data journey was not followed hence resulting in larger DR at report/register (Diff2) than tally sheet/register (Diff1). Diff2 and Diff6 were very similar indicating that data management at the transmission phase does not influence the quality of PNC data (Table 4). Although about half of postnatal indicators performed well in Diff1, there were variations in Diff2. The first indicator (attendance within 48 h), had moderate over-representation, indicating that tally-sheets captured more attendees than those recorded in the registers (Diff1 = 1.30). However, data were extremely over-represented in report forms compared to data entered in the registers. The Diff2 of 3.09 implied that registers had less data compared to what was included in the summary reports. This indicates that reports were not compiled using data from the tally-sheets and cases were not recorded in the registers but somehow summed up in the reports. District performance in PNC differed highly in Diff2 and Diff6. Health workers reported some of the PNC registers and indicators to be difficult to understand.

**Table 4:**
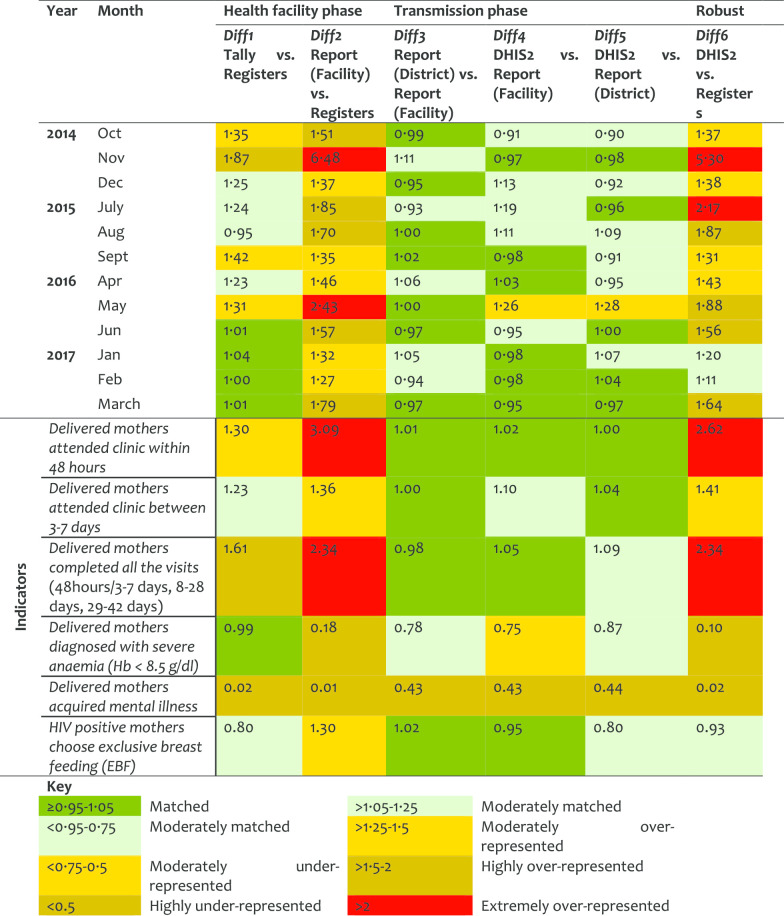
Data accuracy as indicated by difference ratio for the post-natal care service area

*Family planning (FP) service* For the FP service area more data were found in the registers than in the tally-sheets (DR < 1). Comparing earlier years (2014 and 2015) against recent ones (2016 and 2017), an improvement was observed at the transmission phase (Table 5). However, the under-representation of data in tally-sheets did not improve. Overall, half of the indicators in FP services performed quite well with data presenting good matching between tally-sheets, registers and report-forms. An indicator on cervical cancer screening presented a DR less than 1 for Diff1 indicating more data were recorded in the registers than tally-sheets. The screening for breast cancer had a DR of 1.34 for Diff2 indicating that data were compiled in report forms but not indicated in registers. Most of the variations between district performance in FP were observed in Diff1.

**Table 5:**
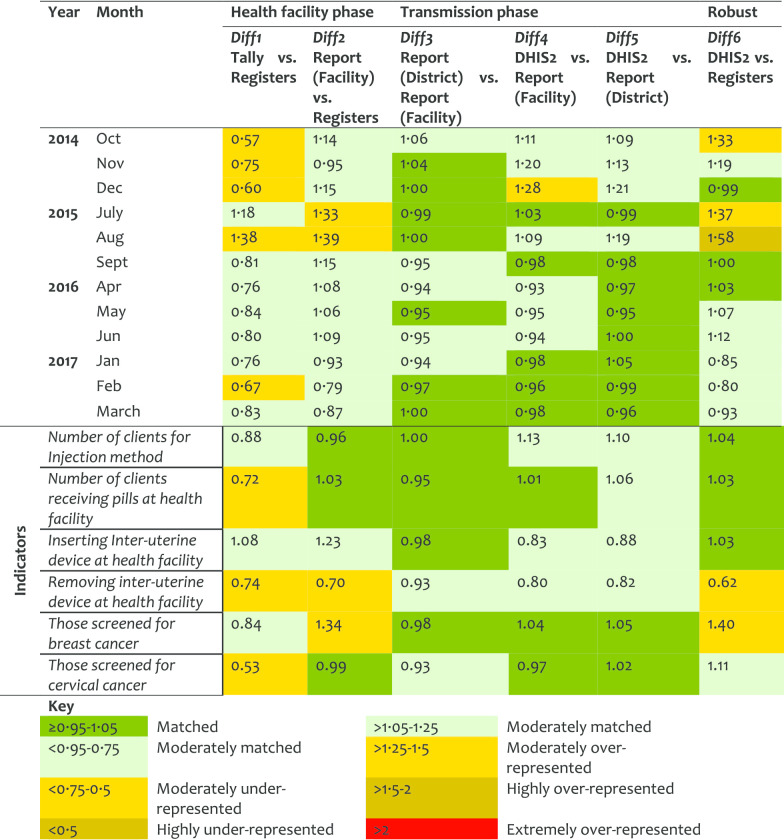
Data accuracy as indicated by difference ratio for the family planning service area

*Outpatient (OPD) service* This service area indicated the highest levels of mismatch in the HF and robust phases. Diff1 showed moderate over-representation in tally-sheets versus registers, which improved significantly over time suggesting adoption on the use of tally-sheets. Extremely large Diff2 and Diff6 values were observed in 2014–2015. It was observed that records in the reports could be up to 5–7-times higher than register records, but was better in 2017 suggesting an improvement in client registration. The transmission phase performed well suggesting moderate manipulation of HF reports before been entered in the DHIS2. The variation in this manipulation between indicator, HFs, and districts is captured by the slight differences observed between Diff2 and Diff6 (Table 6). Individual OPD indicators did not perform well. Data obtained from registers were much less than in the report forms. The indicator of mild/severe anaemia performed worse with DR value indicating a difference of over 6-times between the register records and the report form. Blood smear positive records were corrected in the report forms by inflating values in the copy at the district, with a plus that the changes were documented (Diff3/Diff4 > 1.25). The performance varied between districts, with Kinondoni and Kahama having high levels of data over-representation.

**Table 6:**
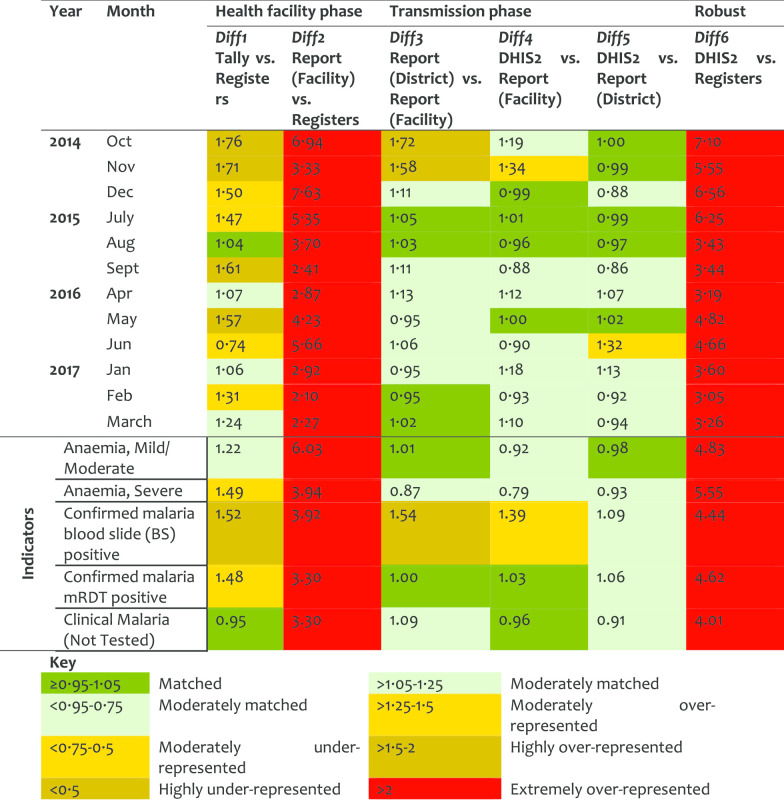
Data accuracy as indicated by difference ratio for the outpatient service area

*Inpatient (IPD) service* IPD was among the service areas that indicated a high level of mismatch between data sources, more particularly over-representation of data in the reports versus records in tally-sheets or registers. This was presented at the HF phase with Diff1 values of > 2 in 2014 and 2016, and Diff2 reporting extreme representation from 2014 to 2017 indicating fewer records in registers versus reported values. Diff1 improved slightly in 2015 and then significantly during 2017 which indicates a better use of tally-sheets. For Diff2 there was no indication of improvement observed during the 4-year period under review (Table 7). At transmission phase, an improvement was observed as the data matched better for the 2016 and 2017. Most of the IPD indicators presented differences between data sources of at least 50%. Data on severe anaemia was extremely over-represented in the tally-sheets and report forms compared to register records (Diff1 = 2.38 and Diff2 = 3.81). This implies that data were not found in registers but were marked in tally-sheets and recorded in the report forms. High difference between registers and reports were observed. There was high variation between district performance in IPD data accuracy mainly in Diff1 and Diff2 with Kinondoni and Dodoma under-utilizing tally-sheets and Mbulu over-representing data in the reports.

**Table 7:**
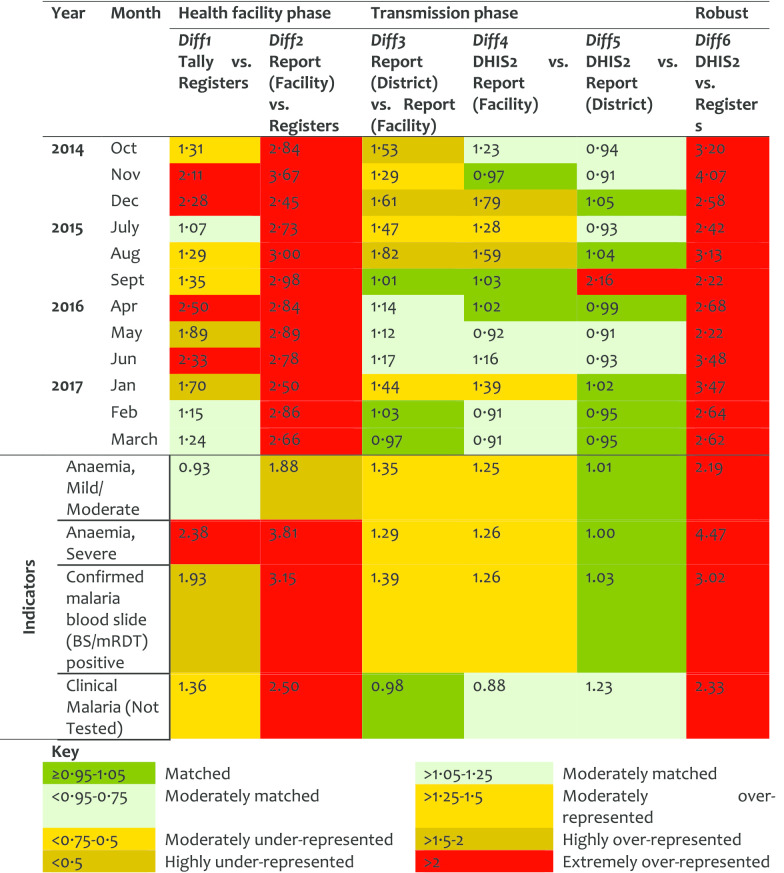
Data accuracy as indicated by difference ratio for the Inpatient service area

*Provider-Initiated Testing and Counselling (PITC) service* In this servicevarea, Diff2 went from moderate (in 2014) to extreme (in 2017) report over-representation indicating weakness in the registration process. PITC reports were highly manipulated during transmission phase before data was entered in DHIS2, marked by large Diff3/Diff4. There were significant variations between HFs and districts for Diff2 and Diff6 (Table 8). Little improvements were observed over the years. Indicator on ‘number of new clients’ was twofold over-represented between the register counts and the reported records and its data was corrected before been entered in DHIS2. Kinondoni, Kibaha and Nkasi showed high levels of data over-representation. Mbulu had matched data for Diff2 but high Diff6 indicating the submitted reports were revised before data were entered in the DHIS2 though, the changes were not documented. Nkasi had the highest Diff4 and Diff5 indicating corrections made during the transmission phase.

**Table 8:**
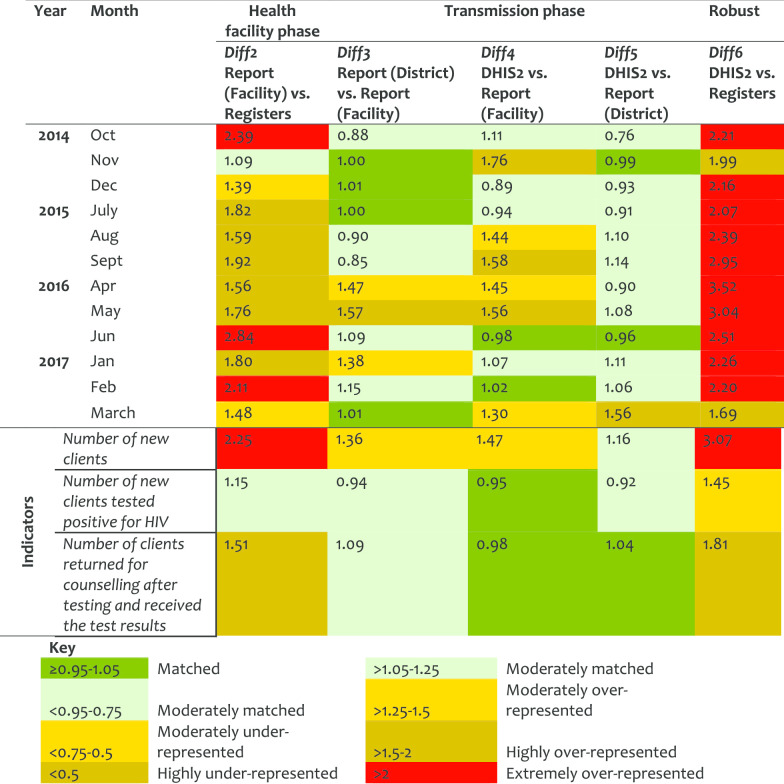
Data accuracy as indicated by difference ratio for the PITC service area

An overall annual pattern indicated a slight improvement on Diff1 (from 1.37 in 2014 to 1.26 in 2017), but a high improvement on Diff2 (from 2.61 in 2014 to 1.70 in 2017). This indicates that although tally-sheets were not fully utilized, the reports were better prepared in 2017 than in 2014. Similarly, there was a marked improvement in values for Diff6 from 2.72 in 2014 to 1.76 in 2017, indicating less variation between register’ records and DHIS2 entries over the years. Data accuracy by HF levels categorized by service areas indicates high Diff2 and Diff6 for hospitals, and in OPD, IPD, ANC, and PNC (Table 9). Data accuracy was observed to vary between HFs even within the districts.

**Table 9:**
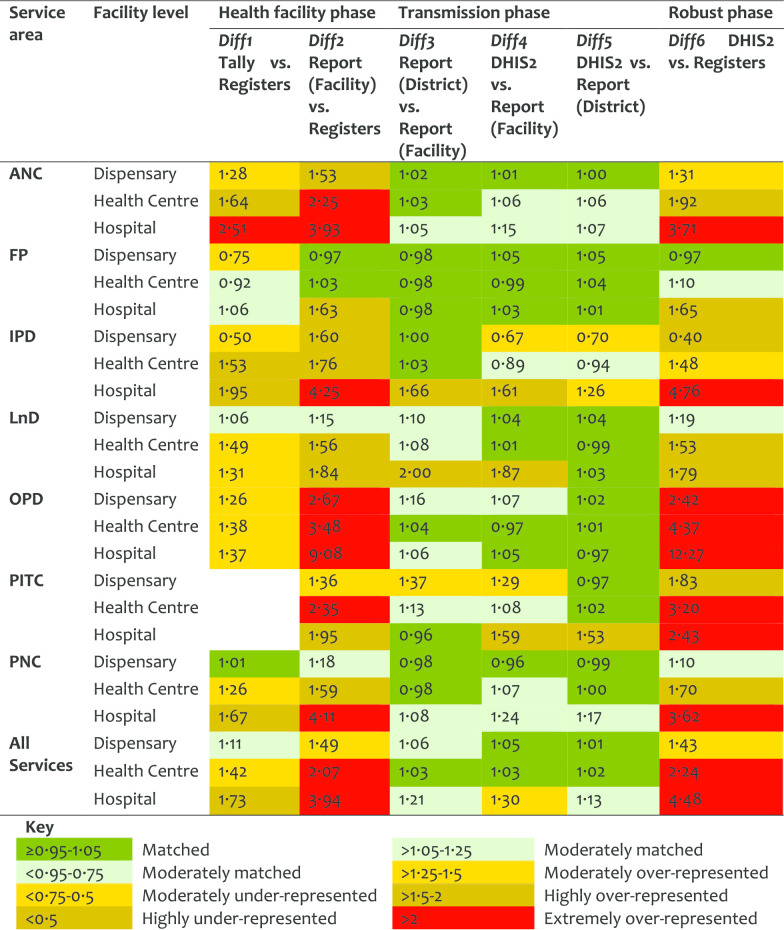
Data accuracy as indicated by difference ratio by HF levels and service area

## Discussion

Registers and report forms were the most commonly available and used HMIS tools in healthcare facilities with high variation between levels of the health system. The urban districts indicated low utilization of HMIS tools than rural ones. The dispensaries, health centres and faith-based owned facilities performed better than hospitals and government-owned facilities. The service area under reproductive and child health performed better than other service areas. The availability rates of report forms submitted to the district offices indicate weakness in the transmission or storage of reports at the district level. Data accuracy varied by district, facility, service area and indicators. The volume of data and complexity in the process of getting indicator data affected the accuracy significantly. Both tool utilisation and data accuracy improved from 2014 to 2017. We observed a reduction in variation on performance within the health system level over time. This is likely to imply improvement and homogeneity in the functioning, resulting in equality on resource allocation and capacity-strengthening programmes. Our methodology assessed the quality while paying attention to health system levels and processes behind routine HMIS data generation to allow systematic thinking along the concerns identified. The approach has facilitated our understanding on the factors related to data quality for each step of the data journey, which is key in designing appropriate interventions.

The variations and inadequate utilization of HMIS tools by facility characteristics have been reported previously in Tanzania and elsewhere [11, 18, 23]. Private-owned facilities, hospitals and healthcare facilities with high client volume are known to significantly affect the quality of HMIS data due to poor adherence in recording procedures, incompleteness and late reporting [3, 5, 6, 9, 13, 28–30]. The performance of urban districts on HMIS tool utilization and data quality has been reported by other studies with inconclusive results [12, 15, 29]. Healthcare facilities in urban areas are assumed to have the infrastructure, sufficient workforce, frequent supervision hence expected to perform better. However, these settings have high workloads due to large catchment population, targeted by multiple programmes and frequent change of human resources resulting in insufficient supervision despite proximity to district offices. These factors are frequently ignored though do highly compromise data quality [2].

There was a high level of mismatch between the register records and those in tally-sheets or report forms, which were highly over-represented. This was common in service areas and healthcare facilities receiving a relatively larger number of clients [29]. These findings highlight difficulties in the utilization of registers when a large number of clients need to be simultaneously attended by the same healthcare provider. Due to the over-burden, inadequate staffing, and the fact that recording is paper-based, it could seem practical and sufficient to tally records in pre-populated sheets than to write patient details in registers, yet not adhering to recording guidelines but be able to produce a report [11, 12]. This phenomenon could explain a significant variation on quality observed between indicators when register counts were compared to subsequent sources [11, 12]. Indicators dealing with a large amount of data and direct client contact, and those associated with dispensing medication/vaccination performed worse [18]. Complex indicators which include a subset of the population served (ANC-5c), time or day specification (PNC-1a and 2), categorization of disease severity had substantial quality issues.

Over-reported routine HMIS data is a challenge in many countries in SSA [18, 22]. Our results indicate a clear reduction in the variation of the mismatch, especially for OPD and IPD service areas from 2014 to 2017. However, a high difference between DHIS2 data and registers’ records still existed particularly for hospital data. The robust phase presented large DR values for many facilities, implying transmitted data are not of good quality. Since the errors originate from the primary source it is difficult to correct them at later stages. A number of studies in other countries have reported similar findings [11, 12, 18, 29]. Our findings indicate that HF reports were manipulated before the data are entered in DHIS2 at the district level. Some studies attribute report manipulation with pressure received from superiors to hide poor service provision, enhance the image of the facility, meet targets or justify the use of medicine [11, 18]. Moreover, it could also be due to inadequacy in supervision and data audits [5, 28]. The variations in data and report management practises within districts, healthcare facilities, and disease programmes have also been documented in other countries [8, 11, 18, 29]. Future research works that document health workers’ opinions on the workload on recording and reporting, and perception on HMIS tools and indicators are necessary to inform and guide intervention in these areas.

The poor quality of the HMIS data and underperformance found in this study and other studies in SSA is likely to be attributed to the combination of multiple factors. These include insufficient human resource with core competence on data management, low motivation and lack of incentives, poor infrastructure, inadequate resources to conduct comprehensive supportive supervision and, lack of standard operating procedures [3, 6, 30–32]. In Tanzania, the majority of the district HMIS focal persons and those dealing with data at the facility level are the same healthcare providers, as a result, they are overburdened, thus key operational problems are not readily identified and remedied timely [11, 29]. For many years, the HMIS in low-income countries have remained paper-based, which is cumbersome, and of uncertain reliability [10]. The DHIS2, recently introduced as a tool to aggregate and process routine facility-based data is expected to facilitate availability, standardization, quality, timely usage, and evidence-based decisions at different levels of the health system [2, 18, 33]. However, DHIS2 is not the magic bullet and will not solve the underlying quality problems currently facing HMIS [18, 34]. Quality assurance and audit should be emphasized at each stage of the *data journey* to detect and address process-loopholes that compromise quality.

Poor data quality has both direct and indirect impacts on the effectiveness and efficiency of the healthcare system. The HMIS aims to produce data that are used to document disease epidemiological patterns and progress towards national goals to improve health programmes. The findings from this study and others [2, 9, 10, 23], indicate that HMIS in low-and-middle income countries are characterized by poor data quality, thus compromising its use in decision making and planning. There is evidence that improved use of routine health data improves the quality of the data as more attention is paid to its demand and usability [10, 35–39]. This means, inadequate data use creates a vicious cycle of inadequate data demands and production of good quality information. It is our expectation that, the findings from this study will stimulate strategic thinking, which consider health system capacity while defining routine HMIS indicators and developing appropriate tools to ensure quality of data generated. Similarly, it is important to strengthen the capacities of the facility and district levels in routine data management and analysis, through introduction of relevant professional cadres.

This assessment has some limitations. First, the study lacked the qualitative component which could address health workers’ perspectives on the causes of the quality issues observed. Second, the data collected from health facility registers and tally sheets were assumed to be correct and no direct observations were done to assess the quality of clinical practice at the health facilities. Lastly, data accuracy metrics were established based on the recording or reporting guidelines as provided by the government. The study did not consider any practice modification improvised by the health worker to facilitate their work.

## Conclusions

In conclusion, investing in HMIS in Tanzania has resulted in improvement in tool utilization and data accessibility in recent years. However, the quality of the routine data is still low. The DHIS2 at the district level inaccurately reflects what exists at the primary facility level (data source). These challenges make HMIS an ineffective tool for monitoring health service performance and as a source of data for planning and decision-making. Findings from this study emphasize the importance of having continuous data quality auditing exercises and innovating strategies that consider the underlying data management processes, indicator types and human resource challenges.

## Data Availability

The datasets used and analysed during the current study are available from the corresponding author on reasonable request.

## Abbreviations

ANC: Antenatal care
DHIS2: District health information system-2
DR: Difference ratio
FP: Family planning
HF: Health facility
HMIS: Health management information system
IPD: Inpatient department
IQR: Interquartile range
LnD: Labour and delivery
OPD: Outpatient department
PITC: Provider initiated testing and counselling

## References

[1] AbouZahr C, Boerma T (2005). Health information systems: the foundations of public health. Bull World Health Organ.

[2] Aiga H, Kuroiwa C, Takizawa I, Yamagata R (2008). The reality of health information systems: challenges for standardization. Biosci Trends.

[3] Ahanhanzo Y, Ouendo E-M, Kpozèhouen A, Levêque A, Makoutodé M, Dramaix-Wilmet M (2015). Data quality assessment in the routine health information system: an application of the Lot Quality Assurance Sampling in Benin. Health Policy Plan.

[4] Chan M, Kazatchkine M, Lob-Levyt J, Obaid T, Schweizer J, Sidibe M (2010). Meeting the demand for results and accountability: a call for action on health data from eight global health agencies. PLoS Med.

[5] Franco L, Fields R, Mmbuji PKL, Posner S, Mboera LEG, Jimmerson A (2003). Situation analysis of infectious disease surveillance in two districts in Tanzania, 2002.

[6] Mavimbe JC, Braa J, Bjune G (2005). Assessing immunization data quality from routine reports in Mozambique. BMC Public Health.

[7] Kimaro H, Sahay S (2007). An institutional perspective on the process of decentralization of Health Information Systems: a case study from Tanzania. Inf Technol Dev.

[8] Sychareun V, Hansana V, Phengsavanh A, Chaleunvong K, Eunyoung K, Durham J (2014). Data verification at health centers and district health offices in Xiengkhouang and Houaphanh Provinces, Lao PDR. BMC Health Serv Res.

[9] Mremi IR, Rumisha SF, Chiduo MG, Mangu CD, Mkwashapi DM, Kishamawe C (2018). Hospital mortality statistics in Tanzania: availability, accessibility, and quality 2006–2015. Popul Health Metr.

[10] Mutale W, Chintu N, Amoroso C, Awoonor-Williams K, Phillips J, Baynes C, Michel C (2013). Improving health information systems for decision making across five sub-Saharan African countries: implementation strategies from the African Health Initiative. BMC Health Serv Res.

[11] Amoakoh-Coleman M, Kayode GA, Brown-Davies C, Agyepong IA, Grobbee DE, Klipstein-Grobusch K (2015). Completeness and accuracy of data transfer of routine maternal health services data in the greater Accra region. BMC Res Notes.

[12] Gimbel S, Micek M, Lambdin B, Lara J, Karagianis M, Cuembelo F (2011). An assessment of routine primary care health information system data quality in Sofala Province, Mozambique. Popul Health Metr.

[13] Teklegiorgis K, Tadesse K, Mirutse G, Terefe W (2016). Level of data quality from Health Management Information Systems in a resources limited setting and its associated factors, eastern Ethiopia. SA J Inf Manag.

[14] Xiao Y, Bochner AF, Makunike B, Holec M, Xaba S, Tshimanga M (2017). Challenges in data quality: the influence of data quality assessments on data availability and completeness in a voluntary medical male circumcision programme in Zimbabwe. BMJ Open.

[15] O’Hagan R, Marx MA, Finnegan KE, Naphini P, Ng’ambi K, Laija K (2017). National assessment of data quality and associated systems-level factors in Malawi. Glob Health Sci Pract.

[16] Chen H, Hailey H, Wang N, Yu P (2014). A review of data quality assessment methods for public health information systems. Int J Environ Res Public Health.

[17] Ahanhanzo YG, Ouedraogo LT, Kpozehouen A, Coppieters Y, Makoutode M, Wilmet-Dramaix M (2014). Factors associated with data quality in the routine health information system of Benin. Arch Public Health.

[18] Bhattacharya AA, Umar N, Audu A, Felix H, Allen E, Schellenberg JRM (2019). Quality of routine facility data for monitoring priority maternal and newborn indicators in DHIS2: a case study from Gombe State, Nigeria. PLoS ONE.

[19] Nshimyiryo A, Kirk CM, Sauer SM, Ntawuyirusha E, Muhire A, Sayinzoga F (2020). Health management information system (HMIS) data verification: a case study in four districts in Rwanda. PLoS ONE.

[20] Ouedraogo M, Kurji J, Abebe L, Labonté R, Morankar S, Bedru KH (2019). A quality assessment of Health Management Information System (HMIS) data for maternal and child health in Jimma Zone, Ethiopia. PLoS ONE.

[21] Nisingizwe MP, Iyer HS, Gashayija M, Hirschhorn LR, Amoroso C, Wilson R (2014). Toward utilization of data for program management and evaluation: quality assessment of five years of health management information system data in Rwanda. Glob Health Action.

[22] Endriyas M, Alano A, Mekonnen E, Ayele S, Kelaye T, Shiferaw M (2019). Understanding performance data: health management information system data accuracy in Southern Nations Nationalities and People’s Region, Ethiopia. BMC Health Serv Res.

[23] Simba DO, Mwangu MA (2005). Quality of a routine data collection system for health: case of Kinondoni district in the Dar es Salaam region, Tanzania. S Afr J Inf Manag.

[24] Rumisha SF, Mboera LEG, Senkoro KP, Gueye D, Mmbuji PK (2007). Monitoring and evaluation of integrated disease surveillance and response in selected districts in Tanzania. Tanzan Health Res Bull.

[25] Mboera LEG, Rumisha SF, Mwanemile EJ, Mziwanda E, Mmbuji PK (2005). Enhancing disease surveillance reporting using public transport in Dodoma District, Central Tanzania. Tanzania Health Res Bull.

[26] Kajeguka AC, Mboera LEG (2003). Information and communication technology: options for strengthening integrated disease surveillance and response at district level in Tanzania. Tanzan Health Res Bull.

[27] Tanzania Demographic and Health Survey and Malaria Indicator Survey. Dar es Salaam, Tanzania and Rockville, Maryland, USA; 2016. https://dhsprogram.com/pubs/pdf/FR321/FR321.pdf. Accessed 14 Apr 2020.

[28] Kihuba E, Gathara D, Mwinga S, Mulaku M, Kosgei R, Mogoa W (2014). Assessing the ability of health information systems in hospitals to support evidence-informed decisions in Kenya. Glob Health Action.

[29] Kasambara A, Kumwenda S, Kalulu K, Lungu K, Beattie T, Masangwi S (2017). Assessment of implementation of the health management information system at the district level in southern Malawi. Malawi Med J.

[30] Adeya G, Bigirimana A, Cavanaugh K, Franco L. Rapid assessment of the health system in Benin: April 2006. Submitted to the US Agency for International Development. 2007. https://www.urc-chs.com/sites/default/files/Benin_Pilot_Test_Assessment_Report.pdf. Accessed 14 Apr 2020.

[31] URT. Independent Verification of Health Service Results Supported by the Health Basket Fund and the Strengthening of Primary Health Care for Results Programme for financial year 2015/16. 2016.

[32] Yazdi-Feyzabadi V, Emami M, Mehrolhassani MH (2015). Health information system in primary health care: the challenges and barriers from local providers’ perspective of an area in Iran. Int J Prev Med.

[33] Maïga A, Jiwani SS, Mutua MK, Porth TA, Taylor CM, Asiki G (2019). Generating statistics from health facility data: state of routine health information systems in Eastern and Southern Africa. BMJ Glob Health.

[34] Maokola W, Willey BA, Shirima K, Chemba M, Armstrong Schellenberg JRM, Mshinda H (2011). Enhancing the routine health information system in rural southern Tanzania: successes, challenges and lessons learned. Trop Med Int Heal.

[35] Braa J, Heywood A, Sahay S (2012). Improving quality and use of data through data-use workshops: Zanzibar, United Republic of Tanzania. Bull World Health Organ.

[36] Wagenaar B, Gimbel S, Hoek R, Pfeiffer J, Michel C, Manuel JL (2015). Effects of a health information system data quality intervention on concordance in Mozambique: time-series analyses from 2009–2012. Popul Health Metr.

[37] Okello G, Molyneux S, Zakayo S, Gerrets R, Jones C (2019). Producing routine malaria data: an exploration of the micro-practices and processes shaping routine malaria data quality in frontline health facilities in Kenya. Malaria J.

[38] Aqil A, Hozumi D, Lippeveld T. Tools for data demand and use in the health sector: Performance of Routine Information Systems Management (PRISM) Tools. MEASURE Evaluation; 2011. https://www.ghdonline.org/uploads/PRISM_DescriptionOfTools.pdf. Accessed 14 Apr 2020.

[39] Mboera L, Rumisha S, Magesa S, Kitua A (2001). Utilisation of health management information system in disease surveillance in Tanzania. Tanzan J Health Res.

